# Supplementary material for: Biomarkers of bleeding and venous thromboembolism in patients with acute leukemia
Source: medRxiv. 2023 Oct 19:2023.10.18.23297216. Preprint. [Version 1] doi: 10.1101/2023.10.18.23297216 (PMC10615001; doi:10.1101/2023.10.18.23297216)
Supplement: Supplement 1 [file media-1.pdf]

Supplementary materials

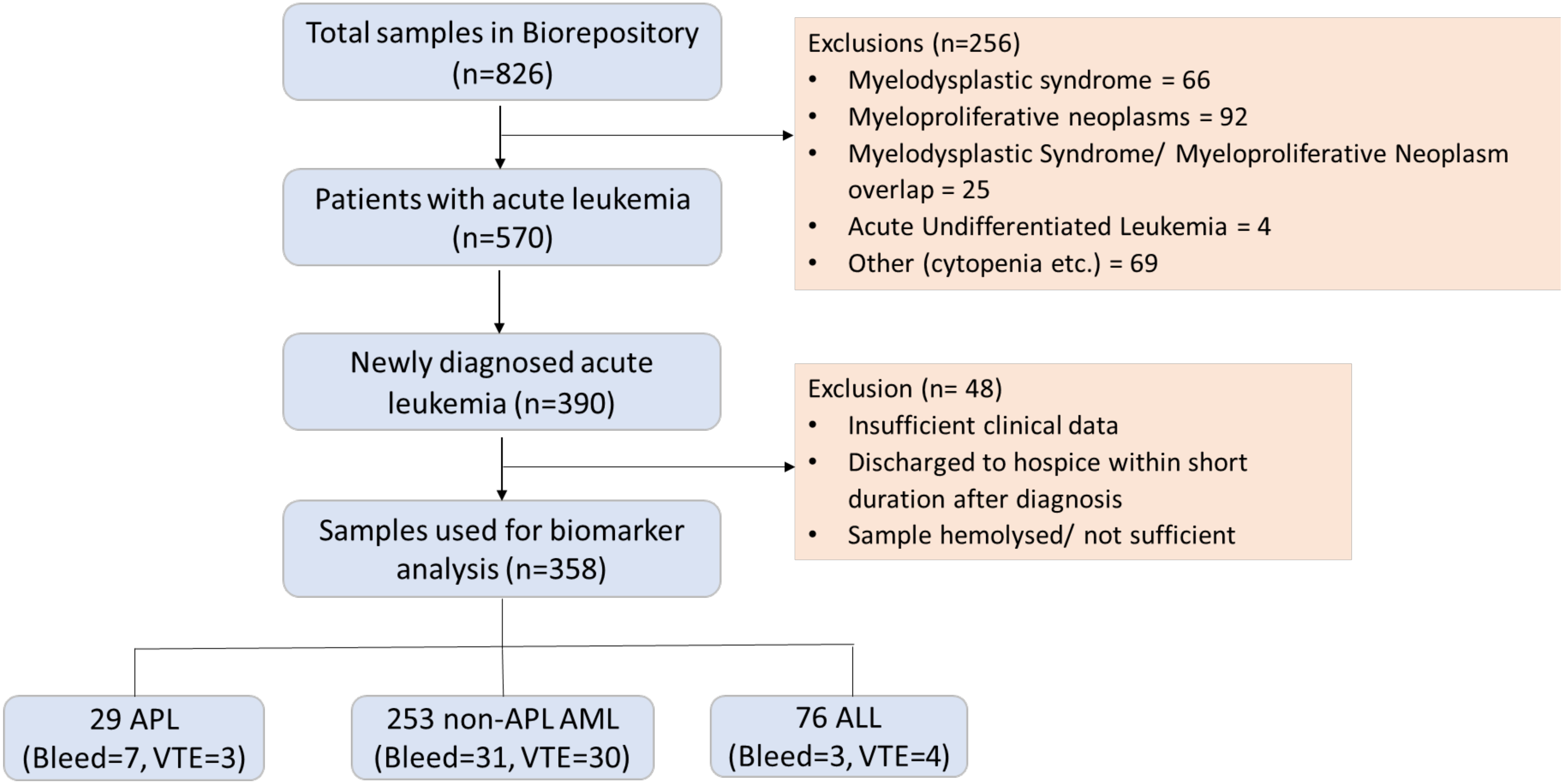

Supplementary Figure 1. Flow chart to demonstrate inclusion and exclusion of patients in the study.

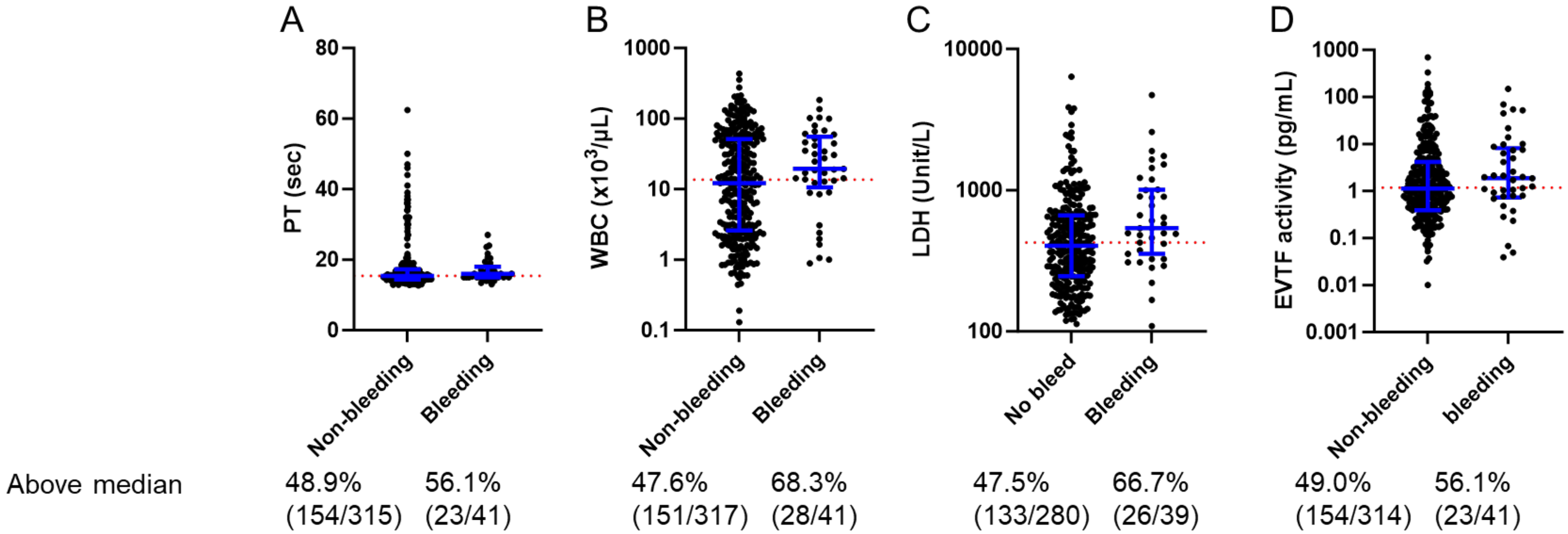

Supplementary Figure 2. Levels of biomarkers between acute leukemia patients with and without bleeding. (A) PT, (B) WBC, (C) LDH and (D) EVTF activity levels in acute leukemia patients with and without bleeding are shown. Blue lines indicate median ± interquartile range. Red dotted lines indicate the median level of all patients.

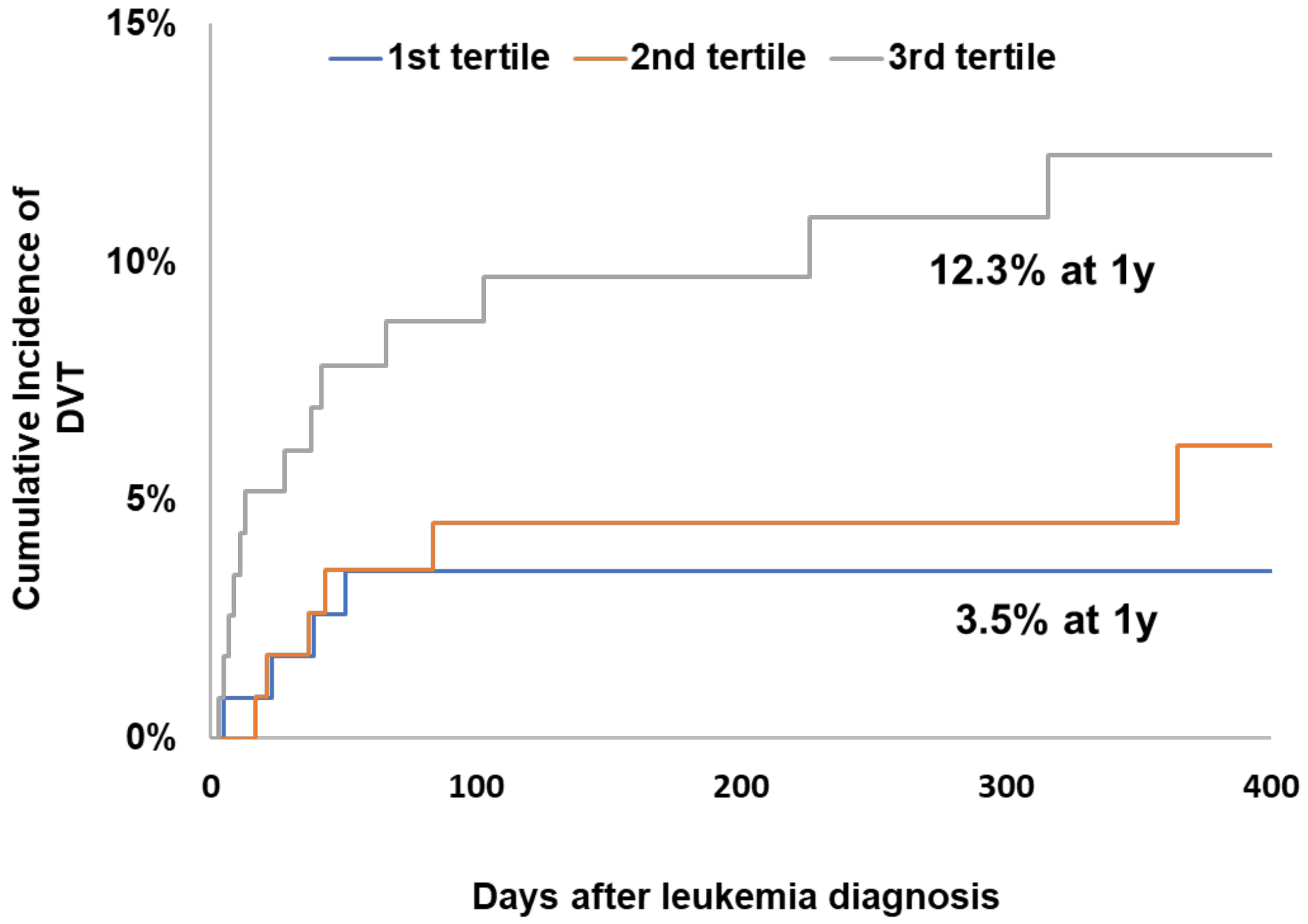

Supplementary Figure 3. Cumulative incidence of deep vein thrombosis by tertiles of plasminogen activator inhibitor-1 levels.

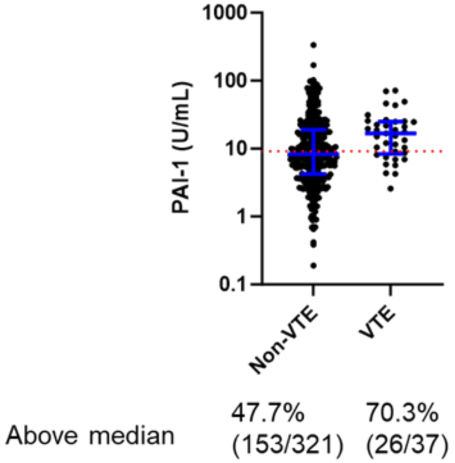

Supplementary Figure 4. Levels of PAI-1 in acute leukemia patients with and without VTE. Blue line indicates median ± interquartile range. Red dotted line indicates the median level of all patients.

Supplementary Table 1. Definitions for disseminated intravascular coagulation, bleeding, and venous thromboembolism outcomes.

Outcome	Definition
Disseminated intravascular coagulation (DIC)	Defined according to the International Society for Thrombosis and Haemostasis (ISTH) criteria and score ≥5 was defined as overt DIC and score <5 as no-DIC:<br>- Platelet count (≥100 x10^3/µL = 0; 50-99 x10^3/µL = 1; <50 x10^3/µL = 2)<br>- Fibrinogen level (≥100 mg/dL = 0; <100 mg/dL = 1)<br>- Prothrombin time (PT) prolongation above the upper limit of normal (ULN) range (<3 seconds = 0; 3-6 seconds = 1; >6 seconds = 2)<br>- D dimer (<2 times ULN normal [480 ng/mL] = 0; 2-4 times ULN (480-960 ng/mL) = 2; >4 times ULN (960 ng/mL) = 3]
Major Bleeding	Defined according to the ISTH classification:<br>- Fatal bleeding<br>- Symptomatic bleeding in critical site/ organ including intracranial, intra-spinal, intraocular, retroperitoneal, intra-articular, pericardial, and intramuscular bleeding with compartment syndrome<br>- Bleeding causing drop in hemoglobin level of ≥2 g/dL or leading to transfusion of ≥2 units of whole blood or red cells
Clinically relevant non major bleeding	Defined according to the ISTH classification:<br>- Bleeding requiring medical intervention<br>- Bleeding resulting in hospitalization<br>- Bleeding prompting face-to-face evaluation<br>- Not fulfilling criteria for major bleeding
Deep vein thrombosis (DVT)	- Acute symptomatic or incidental lower or upper extremity DVT<br>- Catheter related DVT<br>- Acute symptomatic or incidental visceral vein thrombosis<br>- Acute symptomatic or incidental cerebral vein thrombosis
Pulmonary embolism (PE)	- Acute symptomatic PE<br>- Acute incidental PE of the segmental or more proximal pulmonary arterial branches
Superficial vein thrombosis	- Acute symptomatic lower or upper extremity thrombosis involving superficial vein

Supplementary Table 2. Comparisons between acute leukemia patients with high and mid to low EVTF activity.

Parameters	Mid to Low EVTF (n=322) [Median (range)]	High EVTF (n =36) [Median (range)]	P value
WBC count (x 10^3/μL)	11,580 [130-435,910]	59,085 [2,370-171,050]	<0.0001
Peripheral blood blast cell %	56 [0-97]	78.3 [37-96]	<0.0001
D-dimer (ng/mL)	1,138 [135-20,000]	7,884 [230-20,000]	<0.0001
Platelet count (x 10^3/μL)	47,700 [3,400-859,000]	39,600 [7,100-200,000]	0.2488

Supplementary Table 3. Comparisons between APL patients with and without overt DIC.

Characteristics	No-DIC (n=3) [median (range)]	Overt DIC (n=26) [median (range)]	P value
EVTF activity (pg/mL)	1.1 (0.7-3.5)	9.3 (0-188.1)	**0.0353**
PS+EVs (nM)	54.8 (14.4-67.6)	31.0 (7.5-132.7)	>0.9999
cfDNA (ng/mL)	985.3 (868-1,103)	1,144 (799.8-14,766)	NA
H3Cit-DNA complex (ng/mL)	26.9 (0-32.9)	19.5 (0-424.1)	0.9087
PAP (ng/mL)	1,168 (97-15,179)	6,131 (133-15,349)	0.8666
tPA (ng/mL)	0.3 (0-0.9)	0 (0-3.5)	0.0999
PAI-1 (U/mL)	11.0 (7.8-21.2)	16.7 (2.7-87.7)	0.5161
D-dimer (ng/mL)	5,903 (222-6,917)	16,639 (1,376-20,000)	**0.0112**
Fibrinogen (mg/dL)	289 (281-525)	167.5 (60-636)	**0.0367**
PT (sec)	14.5 (13.8-15.5)	17.0 (14.0-27.0)	**0.0435**
PTT (sec)	28 (24-34)	31 (24-200)	0.3612
WBC count (x 10^3/μL)	0.9 (0.8-0.9)	3.3 (0.6-79.8)	**0.0085**
Peripheral blood blast %	17 (2.6-77)	75 (7.5-89)	0.1550
Platelet count (x 10^3/μL)	120 (51.3-131.6)	29.9 (7.1-112.5)	**0.0011**
Creatinine (mg/dL)	0.6 (0.5-1.7)	0.9 (0.6-4.4)	0.3194
Hemoglobin (g/dL)	9.4 (6.9-10.4)	8.6 (3.6-12.4)	0.8283
LDH (Unit/L)	140 (113-166)	325 (133-1,898)	NA
Bleeding (n, %)	0 (0)	7 (26.9)	0.5575
VTE (n, %)	0 (0)	3 (11.5)	>0.9999

Abbreviations: cfDNA, cell-free DNA; EVTF, extracellular vesicle tissue factor; H3Cit-DNA, citrullinated histone H3-DNA; LDH, lactate dehydrogenase; NA, not applicable; PAI-1, plasminogen activator inhibitor-1; PAP, plasmin-antiplasmin complex; PS+EVs, phosphatidylserine-positive extracellular vesicles; PT, prothrombin time; PTT, partial thromboplastin time; tPA, tissue plasminogen activator; VTE, venous thromboembolism; WBC, white blood cell

Supplementary Table 4. Comparisons between non-APL AML patients with and without overt DIC.

Characteristics	No-DIC (n=162) [median (range)]	Overt DIC (n=83) [median (range)]	P value
EVTF activity (pg/mL)	1.1 (0-113)	2.3 (0-4,086)	**0.0001**
PS+EVs (nM)	56.8 (5.9-939.4)	53.6 (0-357.2)	0.2732
cfDNA (ng/mL)	1,164 (338.7-7,783)	1,454 (446.2-20,109)	**0.0003**
H3Cit-DNA complex (ng/mL)	32.1 (0-1,552)	38.2 (0-934)	0.1901
PAP (ng/mL)	5,110 (572.7-43,539)	5,432 (23.6-70,334)	0.5631
tPA (ng/mL)	1.8 (0-38.8)	2.0 (0-19.6)	0.6449
PAI-1 (U/mL)	8.0 (0.2-101.9)	8.9 (0.7-335.4)	0.1146
D-dimer (ng/mL)	732 (135-20,000)	2,182 (624-20,000)	**<0.0001**
Fibrinogen (mg/dL)	456 (181-1,101)	455 (60-795)	0.4014
PT (sec)	15.0 (12.9-47.0)	16.9 (14.0-62.4)	**<0.0001**
PTT (sec)	33 (19-88)	36 (21-61)	**0.0002**
WBC count (x 10^3/μL)	10.3 (0.4-277.3)	42.0 (0.6-435.9)	**<0.0001**
Peripheral blood blast cell (%)	52 (0-95)	72 (2.1-96)	**0.0012**
Platelet count (x 10^3/μL)	66.4 (3.4-862.4)	30.5 (5.7-132.8)	**<0.0001**
Creatinine (mg/dL)	0.9 (0.4-3.2)	1.1 (0.5-3.0)	**0.0343**
Hemoglobin (g/dL)	9.0 (2.9-15.1)	8.7 (3.4-12.8)	0.3687
LDH (Unit/L)	360 (109-3,594)	593.5 (131-5,469)	**<0.0001**
Bleeding (n, %)	14 (8.6)	15 (18.1)	**0.0373**
VTE (n, %)	17 (10.5)	12 (14.5)	0.4054

Abbreviations: cfDNA, cell-free DNA; EVTF, extracellular vesicle tissue factor; H3Cit-DNA, citrullinated histone H3-DNA; LDH, lactate dehydrogenase; PAI-1, plasminogen activator inhibitor-1; PAP, plasmin-antiplasmin complex; PS+EVs, phosphatidylserine-positive extracellular vesicles; PT, prothrombin time; PTT, partial thromboplastin time; tPA, tissue plasminogen activator; VTE, venous thromboembolism; WBC, white blood cell

Supplementary Table 5. Comparisons between ALL patients with and without overt DIC.

Characteristics	No-DIC (n=42) [median (range)]	Overt DIC (n=34) [median (range)]	P value
EVTF activity (pg/mL)	0.7 (0-37.5)	0.5 (0-52.4)	0.1064
PS+EVs (nM)	57.7 (2.7-123.2)	36.3 (4.5-132.4)	**0.0476**
cfDNA (ng/mL)	1,593 (767.4-22,157)	1,677 (116.4-29,325)	0.8407
H3Cit-DNA complex (ng/mL)	18.9 (0-240.1)	19.7 (0-1,607)	0.7649
PAP (ng/mL)	3,563 (125.4-60,381)	7677 (561.5-115,720)	**0.0007**
tPA (ng/mL)	0.5 (0-7.5)	0.5 (0-2.4)	0.3581
PAI-1 (U/mL)	10.0 (1.4-57.7)	8.8 (0.4-69.6)	0.3103
D-dimer (ng/mL)	1,045 (233-11,918)	1,970 (566-16,414)	**0.0024**
Fibrinogen (mg/dL)	401.5 (170.0-856.0)	374.0 (184.0-687.0)	0.5694
PT (sec)	14.4 (12.7-19.2)	15.0 (12.8-21.6)	0.0963
PTT (sec)	32 (24-40)	33 (25-43)	0.2146
WBC count (x 10^3/µL)	18.3 (0.7-204.6)	11.7 (0.1-357.0)	0.3425
Peripheral blood blast cell (%)	60.5 (1.6-94)	64 (2.1-97)	0.8666
Platelet (x 10^3/µL)	80.0 (12.6-377.3)	23.8 (7.9-97.9)	**<0.0001**
Creatinine (mg/dL)	0.9 (0.4-2.1)	0.9 (0.4-2.2)	0.7363
Hemoglobin (g/dL)	9.5 (6.6-17.9)	8.1 (4.6-11.7)	**0.0002**
LDH (Unit/L)	481 (119-2,563)	476.5 (123-6,374)	0.6532
Bleeding (n, %)	0 (0)	3 (8.8)	0.0851
VTE (n, %)	3 (7.1)	1 (2.9)	0.6235

Abbreviations: cfDNA, cell-free DNA; EVTF, extracellular vesicle tissue factor; H3Cit-DNA, citrullinated histone H3-DNA; LDH, lactate dehydrogenase; PAI-1, plasminogen activator inhibitor-1; PAP, plasmin-antiplasmin complex; PS+EVs, phosphatidylserine-positive extracellular vesicles; PT, prothrombin time; PTT, partial thromboplastin time; tPA, tissue plasminogen activator; VTE, venous thromboembolism; WBC, white blood cell

Supplementary Table 6. Characteristics of acute leukemia patients with and without bleeding.

Variable	No Bleeding (n=317)	Bleeding (n=41)	P value
**Age at Diagnosis [median, (range)]**	59 (19-89)	57 (30-75)	0.841
**Sex (n, %)**			
Male	175 (56.1)	28 (60.9)	0.541
**Race (n, %)**			
Non-Hispanic White	232 (74.4)	32 (69.6)	0.502
Black	70 (22.4)	11 (23.9)	
Other/Hispanic or Latino	10 (3.2)	3 (6.5)	
**Body mass index (n, %)**	31 (7.4)	30.1 (8.1)	0.295
**Parameters [median (range)]**			
EVTF activity (pg/mL)	1.1 (0-695.6)	1.9 (0-149.5)	0.111
PS+EVs (nM)	54.6 (0-939.4)	49.9 (4.5-357.2)	0.375
cfDNA (ng/mL)	1,226 (116-29,325)	1,341 (463-14,766)	0.124
H3Cit-DNA complex (ng/mL)	50(0-934)	22 (0-436)	0.141
PAP (ng/mL)	5,014 (24-115,720)	6,251 (1,181-43,539)	0.091
tPA (ng/mL)	0.7 (0-38.8)	1.4 (0-12.6)	0.476
PAI-1 (U/mL)	9.1 (0.4-335.4)	9.5 (0.2-87.7)	0.208
D-dimer (ng/mL)	1,288 (135-20,000)	2,182 (212-20,000)	**0.039**
Fibrinogen (mg/dL)	421 (60-1,101)	441 (60-722)	0.382
PT (sec)	15.1 (12.7-62.4)	16.0 (13.0-27.0)	**0.027**
PTT (sec)	33 (19-200)	33 (24-51)	0.807
WBC count (x 10^3/µL)	12.1 (0.1-435.9)	19.0 (0.9-136.0)	0.148
Peripheral blood blast cell (%)	60 (0-97)	70 (0-91)	0.477
Platelet (x 10^3/µL)	47.7 (3.4-862.4)	35.0 (11.1-285.2)	0.114
Creatinine (mg/dL)	0.9 (0.4-3.2)	0.8 (0.4-4.4)	0.977
Hemoglobin (g/dL)	8.7 (2.9-17.9)	8.5 (3.8-13.6)	0.111
LDH (Unit/L)	404 (110-6,374)	539 (109-4,714)	**0.006**
**History of Bleeding (n, %)**			
Yes	4 (1.3%)	1 (2.2%)	1.261

Supplementary Table 6. Characteristics of acute leukemia patients with and without bleeding. (continued)

Variable	No Bleeding (n=317)	Bleeding (n=41)	P value
**Comorbidities (n, %)**			
None	114 (36.5)	19 (41.3)	0.523
1	78 (25)	8 (17.4)	
≥2	120 (38.5)	19 (41.3)	

Abbreviations: cfDNA, cell-free DNA; EVTF, extracellular vesicle tissue factor; H3Cit-DNA, citrullinated histone H3-DNA; LDH, lactate dehydrogenase; PAI-1, plasminogen activator inhibitor-1; PAP, plasmin-antiplasmin complex; PS+EVs, phosphatidylserine-positive extracellular vesicles; PT, prothrombin time; PTT, partial thromboplastin time; tPA, tissue plasminogen activator; WBC, white blood cell

Supplementary Table 7. Characteristics of acute leukemia patients with and without venous thromboembolism.

Variable	No VTE (n=321)	VTE (n=37)	P value
**Age at Diagnosis [median (range)**	59 (19-89)	54 (20-72)	0.135
**Sex (n, %)**			
Male	185 (58.2)	18 (45.0)	0.113
**Race (n, %)**			
Non-Hispanic White	233 (73.3)	31 (77.5)	0.423
Black	72 (22.6)	9 (22.5)	
Other/Hispanic or Latino	13 (4.1)	0 (0)	
**Body mass index (n, %)**	30.9 (7.6)	31.3 (6.3)	0.426
**Parameters [median (range)]**			
EVTF activity (pg/mL)	1.1 (0-695.6)	1.8 (0-334.2)	0.156
PS+EVs (nM)	53.4 (0-939.4)	60.1 (7.7-326)	0.346
cfDNA (ng/mL)	1,263 (116-29,325)	1,056 (430-6,114)	0.111
H3Cit-DNA complex (ng/mL)	29 (0-1607)	27 (0-404)	0.419
PAP (ng/mL)	5,445 (24-115,720)	4,288 (133-83,099)	0.123
tPA (ng/mL)	0.8 (0-38.8)	0.2 (0-12.6)	0.067
PAI-1 (U/mL)	8.3 (0.2-335.4)	16.7 (2.6-71.6)	**0.002**
D-dimer (ng/mL)	1,383 (135-20,000)	1,154 (233-20,000)	0.943
Fibrinogen (mg/dL)	427 (60-1,100)	378 (98-882)	0.192
PT (sec)	15.2 (12.7-62.4)	15.0 (13.6-24.6)	0.43
PTT (sec)	33 (19-200)	32 (24-50)	0.975
WBC count (x 10^3/μL)	13.2 (0.1-358.1)	18.3 (0.9-435.9)	0.168
Peripheral blood blast cell (%)	60 (0-97)	57 (6-96)	0.884
Platelet (x 10^3/μL)	46 (3-862)	41 (10-353)	0.695
Creatinine (mg/dL)	0.9 (0.4-4.4)	0.9 (0.5-1.8)	0.589
Hemoglobin (g/L)	8.6 (2.9-17.9)	9.0 (3.6-12.8)	0.692
LDH (Unit/L)	424 (109-6,374)	401 (110-5,469)	0.965
**History of VTE (n, %)**			
Yes	14 (4.4%)	3 (7.5%)	0.385

Supplementary Table 7. Characteristics of acute leukemia patients with and without venous thromboembolism. (continued)

Variable	No VTE (n=321)	VTE (n=37)	P value
**Comorbidities (n, %)**			
None	117 (36.8)	16 (40.0)	0.867
1	76 (23.9)	10 (25.0)	
≥2	125 (39.3)	14 (35.0)	

Abbreviations: cfDNA, cell-free DNA; EVTF, extracellular vesicle tissue factor; H3Cit-DNA, citrullinated histone H3-DNA; LDH, lactate dehydrogenase; PAI-1, plasminogen activator inhibitor-1; PAP, plasmin-antiplasmin complex; PS+EVs, phosphatidylserine-positive extracellular vesicles; PT, prothrombin time; PTT, partial thromboplastin time; tPA, tissue plasminogen activator; VTE, venous thromboembolism; WBC, white blood cell

Supplementary Table 8. Biomarkers associated with bleeding and VTE risk in non-APL AML.

Biomarker	Bleeding#		VTE*	
	HR (95% CI)	p-value	HR (95% CI)	p-value
EVTF activity	**2.35 (0.98 - 5.66)**	**0.057**	1.27 (0.58 - 2.77)	0.546
PS + EVs	1.25 (0.61 - 2.56)	0.534	0.89 (0.43 - 1.85)	0.758
cfDNA	1.99 (0.97 - 4.07)	0.061	0.55 (0.28 - 1.08)	0.081
H3Cit-DNA complexes	0.76 (0.40 - 1.46)	0.414	0.54 (0.27 - 1.09)	0.084
PAP	1.30 (0.64 - 2.62)	0.470	0.84 (0.41 - 1.70)	0.623
tPA	0.88 (0.42 - 1.82)	0.726	**0.47 (0.24 - 0.93)**	**0.029**
PAI-1	1.32 (0.65 - 2.66)	0.444	**4.18 (1.47 - 11.87)**	**0.007**
D-dimer	1.32 (0.65 - 2.68)	0.435	1.01 (0.50 - 2.06)	0.979
Fibrinogen	0.66 (0.34 - 1.28)	0.217	0.74 (0.37 - 1.49)	0.402
PT	1.87 (0.85 - 4.13)	0.120	1.55 (0.67 - 3.56)	0.306
PTT	0.66 (0.34 - 1.29)	0.226	1.35 (0.61 - 3.00)	0.454
WBC count	**2.42 (1.04 - 5.62)**	**0.041**	0.94 (0.46 - 1.89)	0.857
Peripheral blood blast cell	1.08 (0.54 - 2.17)	0.827	0.97 (0.45 - 2.08)	0.928
Platelet count	0.56 (0.29 - 1.09)	0.086	0.75 (0.37 - 1.50)	0.411
Creatinine	1.14 (0.49 - 2.63)	0.762	1.10 (0.50 - 2.43)	0.805
Hemoglobin	0.72 (0.38 - 1.39)	0.330	1.44 (0.67 - 3.10)	0.353
LDH	**3.50 (1.34 - 9.17)**	**0.011**	1.08 (0.52 - 2.22)	0.842

Multivariable model was adjusted for age, sex, race/ethnicity,

*Multivariable model was adjusted for age, sex, race/ethnicity, body mass index, history of venous thromboembolism and comorbidities.

Abbreviations: cfDNA, cell-free DNA; EVTF, extracellular vesicle tissue factor; H3Cit-DNA, citrullinated histone H3-DNA; LDH, lactate dehydrogenase; PAI-1, plasminogen activator inhibitor-1; PAP, plasmin-antiplasmin complex; PS+EVs, phosphatidylserine-positive extracellular vesicles; PT, prothrombin time; PTT, partial thromboplastin time; tPA, tissue plasminogen activator; WBC, white blood cell; 95% CI, 95% confidence interval

Supplementary Table 9. Biomarkers associated with intracranial bleeding in patients with acute leukemia

Biomarker	Comparison	Univariable	
		HR (95% CI)	p-value
EVTF activity	Tertile 2 and 3 vs. 1	6.16 (0.82 - 46.34)	**0.0774**
PS + EVs	Tertile 2 and 3 vs. 1	0.82 (0.26 - 2.54)	0.7259
cfDNA	Tertile 2 and 3 vs. 1	2.62 (0.58 - 11.88)	0.2114
H3Cit-DNA complexes	Tertile 2 and 3 vs. 1	1.11 (0.33 - 3.75)	0.8670
PAP	Tertile 2 and 3 vs. 1	0.93 (0.28 - 3.06)	0.9063
tPA	Tertile 2 and 3 vs. 1	0.29 (0.09 - 0.90)	**0.0318**
PAI-1	Tertile 2 and 3 vs. 1	0.73 (0.24 - 2.28)	0.5930
D-dimer	Tertile 2 and 3 vs. 1	2.56 (0.58 - 11.40)	0.2171
Fibrinogen	Tertile 2 and 3 vs. 1	0.27 (0.09 - 0.84)	**0.0234**
PT	Tertile 2 and 3 vs. 1	1.67 (0.37 - 7.42)	0.5023
PTT	Tertile 2 and 3 vs. 1	0.81 (0.25 - 2.69)	0.7357
WBC count	Tertile 2 and 3 vs. 1	1.92 (0.43 - 8.64)	0.3931
Peripheral blood blast cell	Tertile 2 and 3 vs. 1	3.90 (0.51 - 29.97)	0.1912
Platelet count	Tertile 2 and 3 vs. 1	0.85 (0.27 - 2.63)	0.7729
Creatinine	Tertile 2 and 3 vs. 1	3.45 (0.45 - 26.48)	0.2330
Hemoglobin	Tertile 2 and 3 vs. 1	0.55 (0.18 - 1.67)	0.2875
LDH	Tertile 2 and 3 vs. 1	2.14 (0.48 - 9.61)	0.3212

Abbreviations: cfDNA, cell-free DNA; EVTF, extracellular vesicle tissue factor; H3Cit-DNA, citrullinated histone H3-DNA; LDH, lactate dehydrogenase; PAI-1, plasminogen activator inhibitor-1; PAP, plasmin-antiplasmin complex; PS+EVs, phosphatidylserine-positive extracellular vesicles; PT, prothrombin time; PTT, partial thromboplastin time; tPA, tissue plasminogen activator; WBC, white blood cell; 95% CI, 95% confidence interval
